# Seamline Determination Based on PKGC Segmentation for Remote Sensing Image Mosaicking

**DOI:** 10.3390/s17081721

**Published:** 2017-07-27

**Authors:** Qiang Dong, Jinghong Liu

**Affiliations:** 1 Chinese Academy of Science, Changchun Institute of Optics Fine Mechanics and Physics, #3888 Dongnanhu Road, Changchun 130033, China; dongqiang13@mails.ucas.ac.cn; 2 University of Chinese Academy of Science, #19 Yuquan Road, Beijing 100049, China

**Keywords:** remote sensing, image mosaicking, seamline detection, image segmentation, graph cuts, multi-scale morphological gradient (MSMG)

## Abstract

This paper presents a novel method of seamline determination for remote sensing image mosaicking. A two-level optimization strategy is applied to determine the seamline. Object-level optimization is executed firstly. Background regions (BRs) and obvious regions (ORs) are extracted based on the results of parametric kernel graph cuts (PKGC) segmentation. The global cost map which consists of color difference, a multi-scale morphological gradient (MSMG) constraint, and texture difference is weighted by BRs. Finally, the seamline is determined in the weighted cost from the start point to the end point. Dijkstra’s shortest path algorithm is adopted for pixel-level optimization to determine the positions of seamline. Meanwhile, a new seamline optimization strategy is proposed for image mosaicking with multi-image overlapping regions. The experimental results show the better performance than the conventional method based on mean-shift segmentation. Seamlines based on the proposed method bypass the obvious objects and take less time in execution. This new method is efficient and superior for seamline determination in remote sensing image mosaicking.

## 1. Introduction

A large remote sensing image with a wide field of view and high resolution is often required for many applications, such as map-making, disaster management, and military reconnaissance [1,2]. However, the wide field of view and high resolution cannot be captured at the same time because of the limit of the sensor size of the aerial camera. Image mosaicking was used to solve this problem effectively. Image mosaicking is the instrument used to gain a remote sensing image that meets the requirements for both the field of view and resolution using a series of images with overlapping areas. Ideally, the transition in the overlapping region from one image to another should be invisible. Realistically, due to different illumination, exposure parameter settings, depth of field differences, shooting field changes, and other reasons, the overlapping area will inevitably have uneven brightness and geometric misalignment. The problem of brightness unevenness in the mosaicking image can be effectively solved after a series of color corrections, smoothing [3,4,5], and image fusion [6,7,8]. However, the apparent parallax caused by geometric misalignment cannot be solved by the above method. An effective way to solve this problem is to find an optimal seamline in the overlapping region, then take image content respectively on each side. The optimal seamline detection is to find the minimal difference between the two images in the overlapping area, e.g., the intensity difference, gradient difference, and color difference. The geometric misalignment can be eliminated by the above process, and two images can be mosaicked as a large field of view image without apparent parallax.

Since Milgram [9] proposed computer mosaicking technology, finding the optimal seamline to improve the quality of mosaicking image has become an important direction for many scholars to study. Many methods have been proposed to determine the location of the seamline. Yang et al. [10] smoothed the artificial edge effectively through the two-dimensional seam point search strategy. Afek and Brand [11] completed the geometric correction of the image by adding the feature-matching algorithm to the optimal seamline detection process. Fernandez et al. [12] proposed a bottleneck shortest path algorithm to realize the drawing of the aerial photography map by using the absolute value of pixel differences in the overlapping region. Fernandez and Marti [13] subsequently optimized the bottleneck shortest path with a greedy random adaptive search procedure (GRASP) to obtain a superior image. Kerschner [14] constructed an energy function based on color and texture similarity, and then used the twin snakes detection algorithm to detect the position of the optimal seamline. One snake is a profile that moves inside the image, changing its shape until its own energy function is minimal [15]. The twin snakes detection algorithm created start points for two contours on the opposite sides of the overlapping area, which passed through the overlapping area and constantly changed the shape until a new one was synthesized. Soille [16] proposed a mosaicking algorithm based on the morphology and marker control segmentation program, which rendered the seamline along the highlight structure to reduce the visibility of the joints in mosaicking image. Chon et al. [17] used the normalized cross correlation (NCC) to construct a new object function that could effectively evaluate mismatching between two input images. This model determined the horizontal expectations of the largest difference in overlapping region, and then detected the position of the best seamline using Dijkstra’s algorithm. It could contain fewer high-energy pixels in a long seamline. Yu et al. [18] constructed a combined energy function with a combination of multiple image similarity measures, including pixel-based similarity (color, edge, texture), region-based similarity (saliency) and location constraint, and then determined the seamline by dynamic programming (DP) [19]. Li et al. [20] extracted the histogram of oriented gradient (HOG) feature to construct an energy function, then detected the seamline by graph cuts.

Many of the methods described above considered seamline detection as an energy optimization, characterizing the difference between input images in overlapping regions by constructing a special energy function (cost). Image information should be contained in the cost comprehensively, e.g., the color, the gradient, the texture feature and the edge strength, and then find the optimal solution through different optimization algorithms, such as dynamic programming, Dijkstra’s algorithm [21], snake model [15], and graph cuts [22,23]. The core issue is how to avoid the seamline passing through the obvious objects in overlapping area. Owing to the differences of input images, there will be pixel misalignment and color differences near the seamline in the mosaicking image when the seamline passes through the obvious objects. This manifests as the obvious “seam” which can compromise the integrity of objects. Therefore, the seamline should pass through smooth texture region, such as roads, rivers, grass, i.e., background regions, bypassing the obvious objects, such as buildings or cars. It is beneficial to avoid the seamline from passing through the obvious objects to extract the pixel position of objects accurately. Namely, segmentation of the input image is necessary.

Pan et al. [24] proposed an urban image mosaicking method based on segmentation, which determined preferred regions by the mean-shift (MS) algorithm and calculated the color difference as the cost. Firstly, the input images were segmented by the mean-shift algorithm and then the span of every segmented region was computed. Then preferred regions were determined based on the given threshold of the span which was consistent with the size of the largest obvious object in the overlapping regions. Ideally, most of the obvious objects are smaller than the grass or street areas, which are segmented into smaller regions, so that the selected preferred regions do not contain obvious objects, such as buildings, cars, etc. Under the realistic condition, there are some difficulties in the implementation of the method. The result of segmentation strongly depends on the parameters of the mean-shift algorithm, such as bandwidth. In addition, the threshold of the span is related to the size of the objects of the overlapping region, which cannot be completed automatically and needs to be given artificially for each image. Furthermore, the size of the obvious object is not always smaller than the street or the grass, so we cannot accurately extract the preferred regions without obvious objects. This method is too simple to construct the cost map, only considering the color difference, but not the other aspects, e.g., texture or edge. Saito et al. [25] proposed a seamline determination method based on semantic segmentation by training a convolution neural network (CNN). The model can effectively avoid the buildings and obtain a good mosaicking. However, the model needs large-scale image datasets for training, and it is very time consuming.

In this paper, a novel method for seamline determination is presented based on a parametric kernel graph cuts (PKGC) segmentation algorithm [26] for remote sensing image mosaicking. We determine the seamline via a two-level optimization strategy. Object-level optimization is executed firstly. The cost map is weighted by the background regions (BRs) determined by the results of the PKGC segmentation. The cost map contains the color difference, gradient constraint, and texture difference. Then the pixel-level optimization by Dijkstra’s algorithm is carried out to determine the seamline in the weighted cost. This paper is organized as follows: Section 2 describes the novel method of this paper. Section 3 presents the experimental results and the discussion. Section 4 summarizes this paper.

## 2. Methods

Considering the integrity of the mosaicking image, the seamline should pass through flat areas of texture, such as rivers and meadows, bypassing the obvious objects, such as buildings. Therefore, we can set the background regions (BRs) and obvious regions (ORs) with an image segmentation method. The seamline prefers to pass through BRs and round ORs.

**Figure 1:**
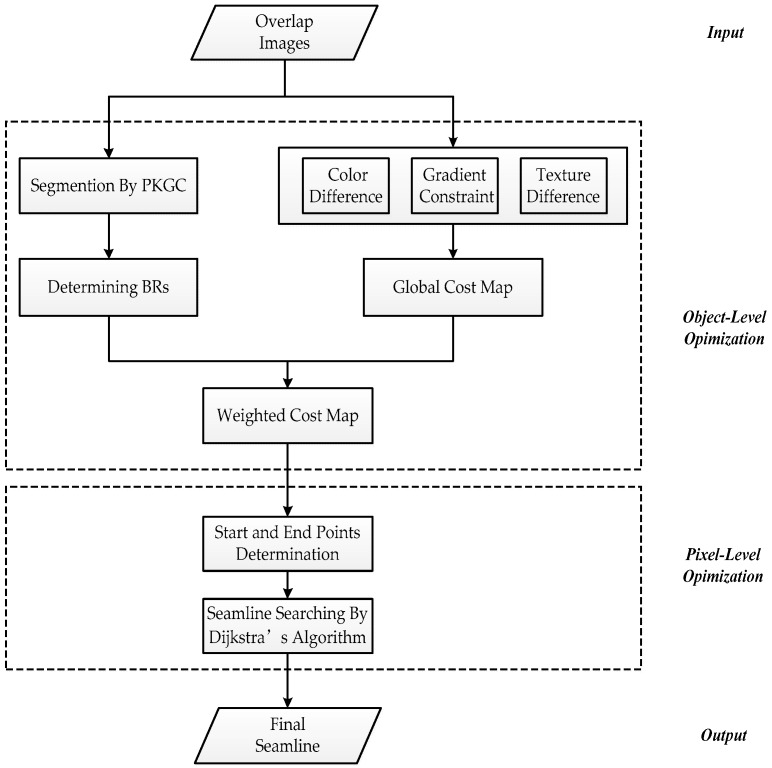
Flowchart of seamline determination.

The corresponding relation of the overlap area of the input images will be determined after pretreatment and registration for input images [27]. Then the seamline can be detected in the overlapping region. Firstly, we determine BRs based on the segmentation by the PKGC algorithm. Then we construct the global cost considering the color difference, the multi-scale morphological gradient (MSMG) constraint, and texture difference. Finally, we determine the pixel position of the seamline by Dijkstra’s algorithm based on the weighted cost map. Figure 1 shows the flowchart of this method.

### 2.1. BR Determination

**Figure 2:**
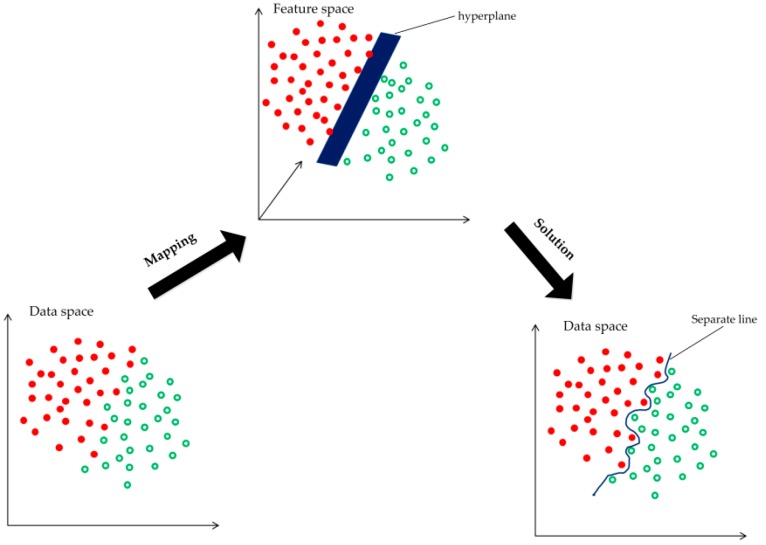
Illustration of nonlinear data separation by mapping to a higher dimensional feature space.

Segmentation by PKGC: The PKGC algorithm borrows from the idea of kernel k-means, and a kernel function ϕ is introduced in the segmentation. The image data is implicitly mapped into the high-dimensional feature space. This makes it possible to highlight the slight difference between the image data, so that the original data, which cannot be divided, is linearly separable (or approximately linear), as Figure 2 shows. This is helpful to construct the piecewise constant model (PCM) containing only the dot product operation and the unsupervised segmentation function. By Mercer’s theorem, any continuous, symmetric and positive semi-definite kernel function can be expressed as a dot product of higher dimensional space without knowing the mapping.

The kernel graph cuts model needs to set $N_{seg}$ marks for the $N_{seg}$ regions firstly, and then every pixel of the image is assigned a mask. Finally, determine which region that each pixel belongs to according to the mark. The segmentation by graph cuts method in the kernel-induced space is transformed into finding a mark allocation scheme to minimize the energy function. The energy function contains two items: the first is the kernel-induced distance term, which is used to estimate the deviation between the mapped data in each region of the PCM model, and the second is the smoothing term which can smooth adjacent pixels. The energy function is as follows:

(1) $$E_{K}(\{\mu_{l}\},\lambda )={\sum_{l\in L}\sum_{p\in R_{l}}J_{K}(\phi (\mu_{l})-\phi (I_{p}))}^{2}+\alpha \sum_{\{p,q\}\in N}r(\lambda (p),\lambda (q))$$

where $E_{K}$ is the non-Euclidean distance between the region’s parameter and the observations. $\mu_{l}$ is the PCM parameter of region $R_{l}$, which can be acquired by the k-means clustering algorithm. λ is the indexing function assigning a label to the pixel. l is the label of the segmentation region. L is the number of segmentation regions. $R_{l}$ means the region of label l. ϕ is the nonlinear mapping from image space I to the higher dimensional feature space J. The commonly-used function is the radial basis function (RBF), $K(y,z)=\exp(-||y-z|{|}^{2}/\sigma^{2})$. p and q represent two adjacent pixels. r(λ(p),λ(q)) is the smoothing function, $r(\lambda (p),\lambda (q))=\min\{c,|\mu_{\lambda (p)}-\mu_{\lambda (q)}{|}^{2}\}$, where c is constant. α is a non-negative factor used to weigh the two terms. Then introducing the kernel function:

(2) $$K(y,z)=\phi {\left(y\right)}^{T}•\phi (z),\;\forall (y,z)\in I^{2}$$

where “•” is the dot product in the feature space. The non-Euclidean distance of the feature space can be expressed as follows:

(3) $$\begin{array}{ll} J_{K}(I_{p},\mu ) & =||\phi (I_{p})-\phi (\mu )|{|}^{2}\\ & =K(I_{p},I_{p})+K(\mu ,\mu )-2K(I_{p},\mu ),\;\mu \in {\left\{\mu_{l}\right\}}_{1\leq l\leq N_{reg}} \end{array}$$

Then, substitution of Equation (3) into Equation (1) results in the expression:

(4) $$E_{K}(\{\mu_{l}\},\lambda )={\sum_{l\in L}\sum_{p\in R_{l}}J_{K}(I_{p},\mu_{l})}^{2}+\alpha \sum_{\{p,q\}\in N}r(\lambda (p),\lambda (q))$$

Clearly, the solution of Equation (4) depends only on the regional parameters ${\left\{\mu_{l}\right\}}_{l=1,2,\cdots ,N_{reg}}$and the indexing function λ. The iterative two-step optimization method is used to minimize the function. Firstly, fix the labeling results (image segmentation) and update the current statistics region parameter. Optimize $E_{K}$ for the given kernel function. Then search for optimal labeling results (image segmentation) using the graph cuts iteration base on the region parameter obtained above.

Determining BRs: The image can be segmented into the foreground obvious objects regions and the background regions. Two input images are segmented using the PKGC algorithm independently. The BRs determine the intersection of the segmentation results of the left image and the right image. The remaining regions of overlapping area are regarded as ORs, i.e., the union of the segmentation results.

### 2.2. Constructing the Energy Function

We consider the following steps to construct a more accurate energy function. Firstly, calculate the global energy function C(x,y) and then obtain the weighted cost D(x,y) weighted by the BRs. Let the compound image *I* be the overlapping region of the input left image $I_{1}$ and the right image $I_{2}$. The global energy function C(x,y) of pixel (x,y) contains several aspects as follows:

#### 2.2.1. Color Difference

Color difference is the most common energy function in seamline detection for image mosaicking. We calculate the difference in the HSV (hue, saturation, value) color space instead of the common RGB space. The color difference $\delta_{c}(x,y)$ is defined as follows:

(5) $$\delta_{c}(x,y)=\omega \left|V_{1}(x,y)-V_{2}(x,y)\right|+(1-\omega )\left|\left(S_{1}(x,y)-S_{2}(x,y)\right)\right|$$

where $V_{1}(x,y)$ and $S_{1}(x,y)$ is the intensity values of pixel (x,y) in the V and S channels of the HSV space of the left image $I_{1}$. Weight coefficient ω∈[0,1] is used to balance the effects of V and S channels, and equals 0.95 in this paper. Similarly, $V_{2}(x,y)$ and $S_{2}(x,y)$ express analogous meaning.

#### 2.2.2. MSMG Constraint

**Figure 3:**
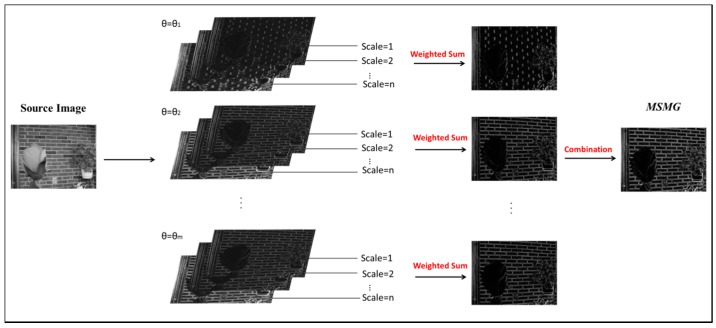
Demonstration of constructing the multi-scale morphological gradient.

In the image morphological processing, the structural element is a common tool for image feature extraction and the structural element with different shapes can extract different image features. Furthermore, changing the size of the element can be extended to the multi-scale space [28]. The gradient can represent the sharpness of an image [29,30]. The comprehensive gradient feature will be extracted by the multi-scale morphological gradient operator [31,32]. In this paper, we propose a novel multi-angle linear structural element to extract the multi-scale morphological gradient (MSMG), extracting the multi-scale gradient of each angle and then combining them into the multi-scale morphological gradient, as Figure 3 shows. The details of this method are given as follows:

Firstly, construct the multi-scale element:

(6) $$SE_{j}^{\theta }=\underset{j}{\underset{︸}{SE_{1}^{\theta }⊕SE_{1}^{\theta }\cdots ⊕SE_{1}^{\theta }}},\;j\in \{1,2,\cdots ,n\},\;\theta \in \{\theta_{1},\theta_{2},\cdots ,\theta_{m}\}$$

where $SE_{1}^{\theta }$ is the basic linear structural element with length l and angle θ, and n is the sum of scales.

Then the gradient feature $G_{j}^{\theta }$ with scale j and angle θ will be extracted by the above operator. Let image I=f(x,y).

(7) $$G_{j}^{\theta }(x,y)=f(x,y)⊕SE_{j}^{\theta }-f(x,y)⊖SE_{j}^{\theta }$$

where ⊕ and ⊖ is the morphological dilation and erosion respectively, which are defined as:

(8) $$f⊕SE=\underset{\left(u,v\right)}{\max}(f(x-u,y-v)+SE(u,v))$$

(9) $$f⊖SE=\underset{\left(u,v\right)}{\min}(f(x+u,y+v)-SE(u,v))$$

where (x,y) is the coordinate of the pixel in the image, and (u,v) is the coordinate of the structural element.

According to the above definition, the maximum and minimum gray value of the local image region can be obtained by dilation and erosion operators, respectively. The morphological gradient is defined as the difference of the dilation and erosion, which can extract the local information effectively. Meanwhile, we can obtain more comprehensive information by changing the scale and angle of the linear structural element. The large scale indicates the gradient information within long distances, while the gradient information with short distances is indicated by the small scale. Angle 0° indicates the horizontal gradient information, and angle 90° indicates the vertical gradient information.

Finally, gradients of all scales and all angles are integrated into the multi-scale morphological gradient *MSMG*.

(10) $$MSMG(x,y)=\sqrt{\frac{2\sum_{\theta =1}^{m}{\left(\sum_{j=1}^{n}\varepsilon_{j}\cdot G_{j}^{\theta }(x,y)\right)}^{2}}{m}}$$

where *m* is the number of angle θ, and $\theta =\{0^{{}^{\circ}},45^{{}^{\circ}},90^{{}^{\circ}},135^{{}^{\circ}}\}$ in this paper, i.e., m=4. n is the numbers of scales, and n=5 in this paper. $\varepsilon_{j}$ is the weight of gradient in scale j, $\varepsilon_{j}=1/(2\times j+1)$.

The MSMG constraint $\delta_{g}(x,y)$ of pixel (x,y) is defined as:

(11) $$\delta_{g}(x,y)=\max(MSMG_{1}(x,y),MSMG_{2}(x,y))$$

where $MSMG_{1}(x,y)$ and $MSMG_{2}(x,y)$ are the multi-scale morphological gradients of the pixel (x,y) in the left $I_{1}$ image and the right image $I_{2}$.

#### 2.2.3. Texture Difference

In this paper, we calculate the image entropy of the 3 × 3 neighborhood of the pixel to represent the local texture features of the image. We iterate through all pixels using the entropy filter of size 3 in the implementation. Image entropy is the measure of data randomness in an image’s gray histogram, which is calculated by:

(12) $$I^{t}=-\sum p\log_{2}p$$

where p is the total number of histograms of image I. The detail texture information cannot be represented by image entropy due to only being based on the frequency of neighborhood data regardless of the intensity contrast. Considering the amalgamating of the variations of illumination and contrast in the image, it is considered adequate to regard image entropy as the coarse representation of texture features. The texture difference $\delta_{t}$ is defined as following.

(13) $$\delta_{t}(x,y)=abs(I_{1}^{t}(x,y)-I_{2}^{t}(x,y))$$

where $I_{1}^{t}(x,y)$is the image entropy of the 3 × 3 neighborhood of the pixel (x,y) in the image, similar to $I_{2}^{t}(x,y)$.

The global cost C(x,y) is combined by the above three terms.

(14) $$C(x,y)=(\delta_{c}(x,y)+\delta_{g}(x,y))\times \delta_{t}(x,y)$$

Then we weight C(x,y) by the BRs obtained in Section 2.1 to obtain the weighted cost D(x,y).

(15) $$D(x,y)=\{\begin{array}{cc} \upsilon C(x,y), & f(x,y)\in BRs\\ C(x,y), & otherwise \end{array}$$

where υ is the weight coefficient of BRs, 0<υ<1. The seamline should preferentially pass through the BRs around the obvious objects, so we give a small weight value for the BRs.

### 2.3. Pixel-Level Optimization

**Figure 4:**
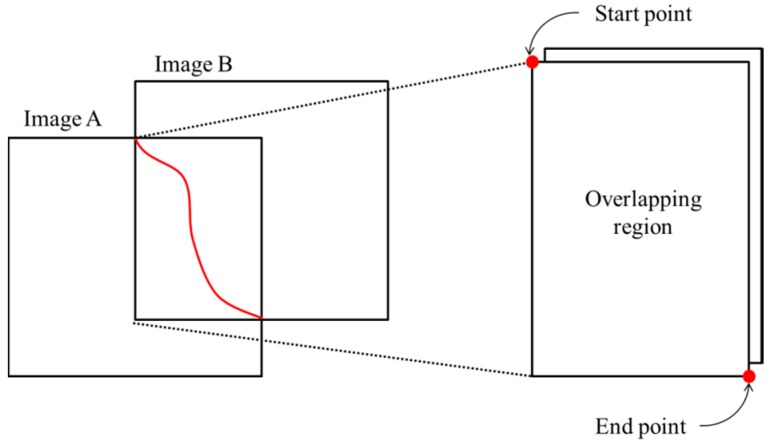
Determining overlapping region and start/end point.

The purpose of the pixel-level optimization is to optimize the location of the seamline in the local area. As shown in Figure 4, the overlap area of the image can be gained after determining the relation of the input images, and the intersection of the input images edges is determined as the start and end point of the seamline. Then we detect the shortest path based on the weighted cost from the start point to the end point.

**Figure 5:**
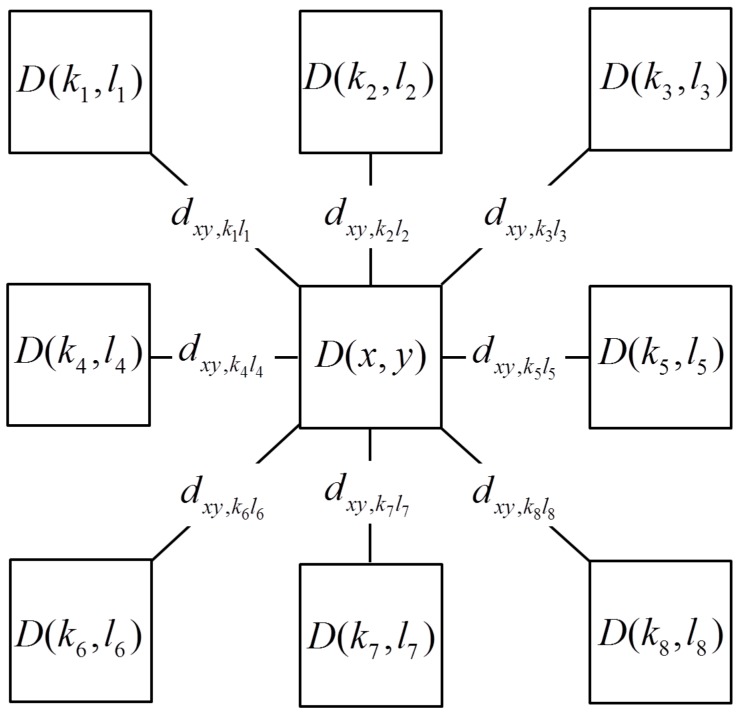
Node and neighbor nodes.

Dijkstra’s algorithm is a global optimization technique to find the shortest path between the two nodes in the graph. Each pixel in the overlapping region is regard as one node which has eight neighbor nodes. As shown in Figure 5, the local cost is calculated based on the cost difference of neighbor nodes when detecting the shortest path using Dijkstra’s algorithm. Let (x,y) be one node and $\left(k_{m},l_{m}\right)$ be a neighbor node of this node. The local cost of these two nodes is defined as:

(16) $$d_{xy,k_{m}l_{m}}=\left|D(x,y)-D(k_{m},l_{m})\right|,\;m=1,2,\cdots ,8$$

where D(x,y) and $D(k_{m},l_{m})$ are the weighted cost of pixel (x,y) and $\left(k_{m},l_{m}\right)$. Let NBR(x,y) be all adjacent nodes of the node (x,y). cost(x,y) and $cost(k_{m},l_{m})$ represent the global minimum cost from the start node to pixel (x,y) and $\left(k_{m},l_{m}\right)$, respectively.

(17) $$cost(x,y)=\min(d_{xy,kl}+cost(k_{m},l_{m});(k_{m},l_{m})\in NBR(x,y))$$

### 2.4. Multi-Image Seamline Detection

**Figure 6:**
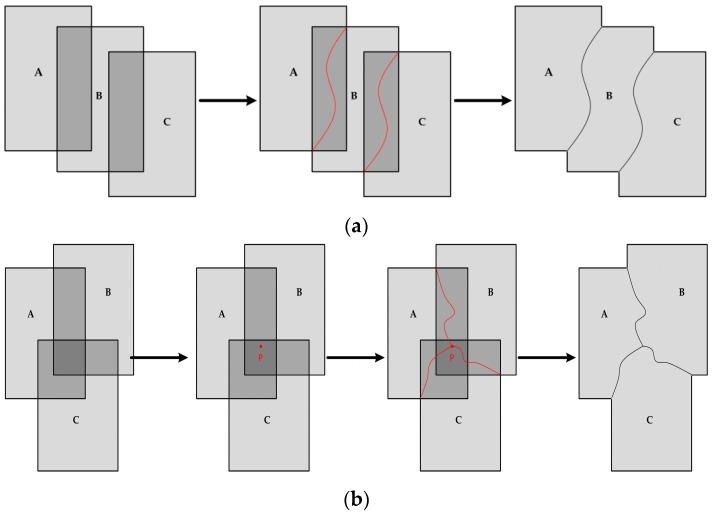
Seamline detection: (**a**) two-image overlap; (**b**) multi-image overlap.

We introduce the method of seamline determination in a two-image overlapping region and can obtain a panoramic image with a wide field of view and high resolution by mosaicking a set of images using this method. As shown in Figure 6a, in the process of multi-image mosaicking, we hope that there is no multi-image overlap, i.e., the overlapping regions are all two-image overlap. In practical applications, the regions are always multi-image overlap. Figure 6b shows an illustrative example where the overlapping region is overlapped by three input images A, B, and C. The traditional method is to detect the seamline just between each of the two images, named frame-to-frame [33]. To mosaic multiple images more accurately, we propose a new optimization strategy to detect seamlines for multi-image overlap. Firstly we find the point p(x,y) which is the weighted cost minimum in the setting rectangle. The center of this rectangle is the center of the multi-image overlapping region and its length and height are a quarter of the overlapping region. Then we detect the seamlines from the point p(x,y) to the joint points of AB, AC, and BC. Finally, the panoramic image is mosaicked based on the optimal seamlines.

## 3. Experiment and Discussion

The method we proposed was implemented by C++ (VC 10.0) and Matlab (2012b), combined. A desktop computer with an Intel Core i5 CPU at 3.2 GHz and 4 GB of memory was used. Experiments were conducted using three sets of orthoimages. In order to improve the efficiency, we built one pyramid level that was built by a reduction factor of three with a 3 × 3 average filter. Five sets of experiments were carried out to verify the effectiveness and superiority of our method.

### 3.1. Experiment of Image Segmentation

An image segmentation comparison was performed for Image Set 1 to verify the advantages of the BRs obtained by the PKGC segmentation we introduced in Section 2.1. Mean-shift was also carried out in the pyramid with parameter $\left(h_{s},h_{r},M)=(6,5,30\right)$, where $\left(h_{s},h_{r}\right)$ is the bandwidth and M is the smallest feature size. The weight υ of BRs was 0.1.

**Figure 7:**
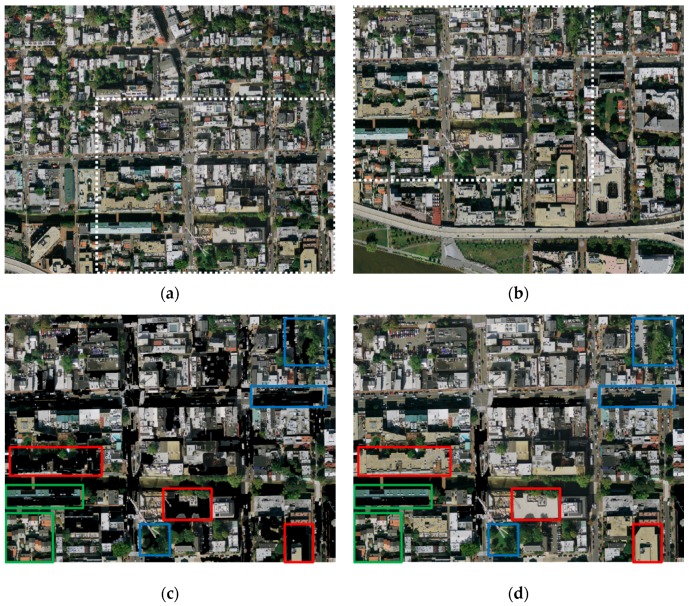

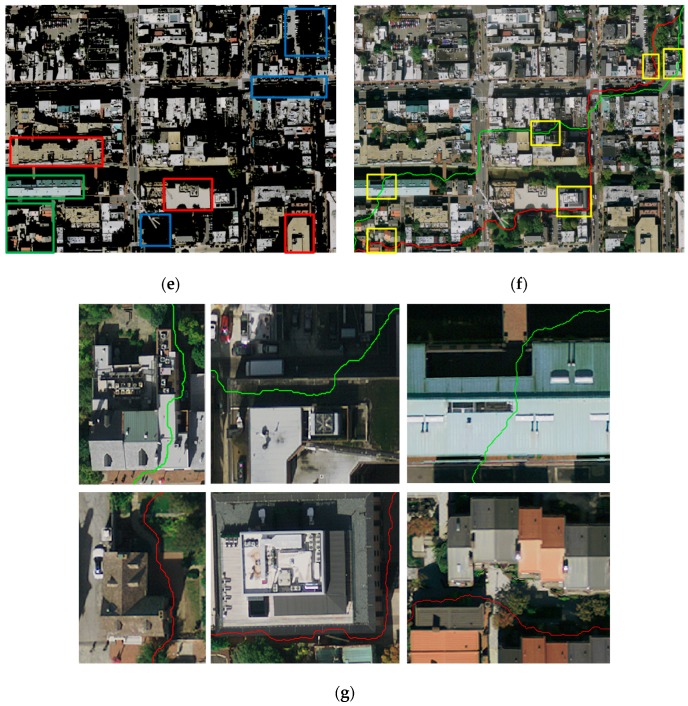
Experimental results of image set 1: (**a**) the left image; (**b**) the right image; (**c**) mean-shift segmentation result ($S_{T}$ = 50); (**d**) mean-shift segmentation result ($S_{T}$ = 80); (**e**) PKGC segmentation result; (**f**) seamlines by Pan’s method (green line) and ours (red line); (**g**) details marked rectangles in (**f**).

The results of image segmentation are displayed in Figure 7. Figure 7a,b shows the left image and the right image, respectively, whose size is 3800 × 3200 pixels and the dotted rectangle indicates the overlapping areas. The input images are segmented by mean-shift segmentation proposed in Pan’s method. The span of regions that are larger than the given threshold $S_{T}$ will be extracted as the BRs. $S_{T}$ is the threshold of the span and usually equals the maximum size of significant targets. We test a set of different values ranging from 30 to 200 for determining the appropriate threshold $S_{T}$. When $S_{T}$ is small, the BRs can be extracted, but the obvious objects with the larger size will be treated as BRs, as the red rectangles show in Figure 7c ($S_{T}$ = 50). When $S_{T}$ is larger, it can increase the extraction of obvious objects, but at the same time the background area cannot effectively be separated and will be regarded as ORs, as the blue rectangles show in Figure 7d ($S_{T}$ = 180). When the background region is too small, or the object is too large, it cannot be effectively segmented no matter what value $S_{T}$ is equal to, as the green rectangles show in Figure 7c,d. Figure 7e shows the segmentation result based on the PKGC algorithm. The objects and background can be segmented effectively by our method as the rectangles show. Therefore, the BRs obtained by our method are more accurate. The BRs getting by Pan’s method depend on the value of the span threshold $S_{T}$. We must give a suitable $S_{T}$ for every input image manually, which is apparently difficult. If the size of the object is larger than the size of the maximum background area, $S_{T}$, which can distinguish the large objects and the backgrounds, is non-existent, so the seamline passes through the obvious objects. Figure 7f shows the seamlines based on Pan’s method ($S_{T}$ = 80) and our method. The green line is Pan’s method and the red line is ours. Figure 7g shows the enlarged details of the yellow rectangle regions in Figure 7f. We can see that the seamline by the proposed method successfully bypasses all of the obvious objects, but the seamline by Pan’s method passes through the buildings.

### 3.2. Experiment of Energy Function

**Figure 8:**
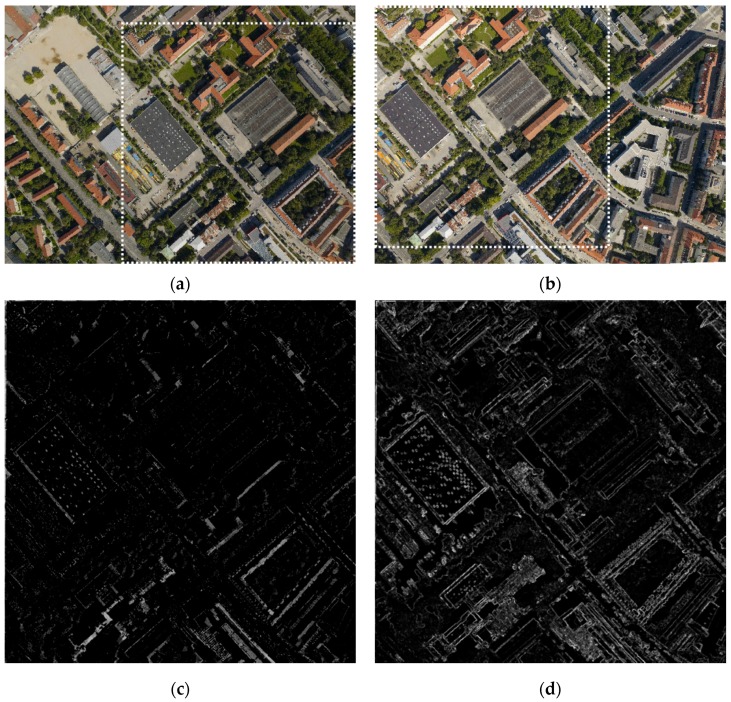

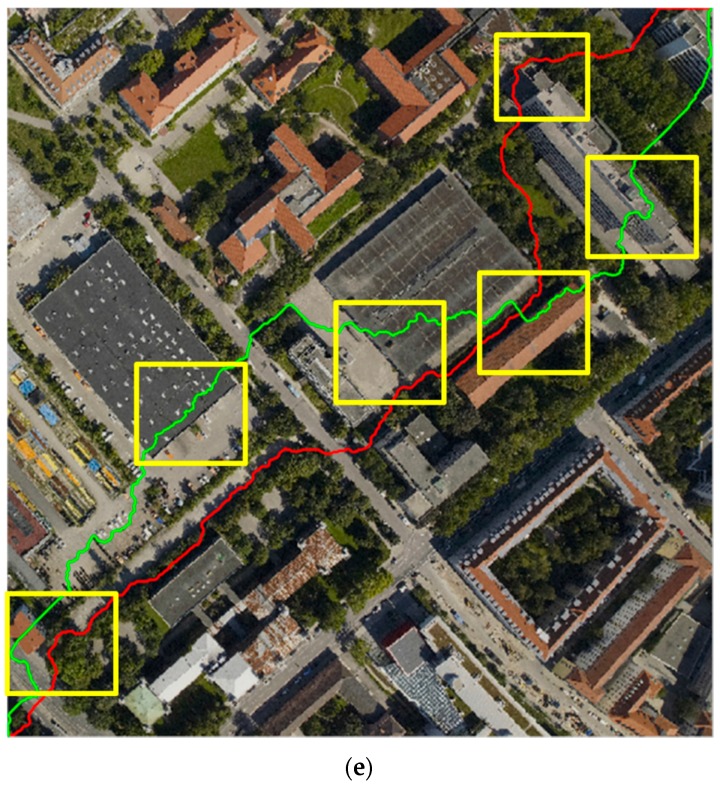
Experimental results of image set 2: (**a**) the left image; (**b**) the right image; (**c**) Pan’s cost; (**d**) our cost; (**e**) seamlines based on Pan’s cost (green line) and ours (red line).

In order to verify the rationality of the energy function proposed in Section 2.2, we compared our cost and Pan’s cost, then analyzed the results of the experiment for Image Set 2. The weight parameter of the color difference ω = 0.95. In the process of calculating the MSMG, angle θ = $\left\{0^{{}^{\circ}},45^{{}^{\circ}},90^{{}^{\circ}},135^{{}^{\circ}}\right\}$ and the numbers of scales n = 5. The results of the energy function are displayed in Figure 8. Figure 8a,b shows the left image and the right image, respectively, whose size is 1900 × 1400 pixels, and the dotted rectangle indicates overlapping areas. The absolute value of the gray difference of the corresponding pixel is the same as the cost of the overlapping region directly, as Figure 8c shows. Although this method is simple, its stability is not strong, especially when the brightness difference of the overlapping region is small. Comprehensively considering the gray, texture, and edge information of the image, we proposed a new method to construct the energy function. Especially, the MSMG we proposed in Section 2.2.2 can extract the gradient information in scale space effectively, so the energy function is more accurate. As Figure 8d shows, the edge and the texture information are extracted comprehensively and clearly. Figure 8e shows the location of seamlines based on the cost of Pan’s method and our method. The green line is Pan’s method and the red one is our method. The seamline based on our method can effectively avoid significant target edges and regions with complex textures. The cost we proposed can effectively reflect the salient information of the images. It can still extract the edge and the texture of the image, even though the gray values of input images are close. Contrarily, the cost based only on color difference is easily influenced by the brightness of the images. When the difference of brightness is too small, the obvious objects will be submerged in the BRs and the seamline will pass through the objects.

### 3.3. Analysis of Time-Consumption

For Image Set 1 and 2, we determined the seamline using Chon’s method [17], Li’s method [20], Pan’s method [24], and the proposed method, respectively, then recorded the time-consumption and the number of obvious objects passed through. Table 1 shows the performance comparison of different methods. To compute the NCC in Chon’s method takes much time, and determining the seamline by iteration is intricate. Thus, the time-consumption is very large. Since the HOG feature of the 11 × 11 neighborhood is computed for every pixel, the calculation quantity is sizable. The main time-consumption of Pan’s method is to determine preferred regions, which need to compute the span of every segmentation region. The more segmentation regions mean the more time-consumption. The proposed method takes less time than other methods. Meanwhile the seamline does not pass through any obvious object.

### 3.4. Analysis of Suppressing Noise

**Figure 9:**
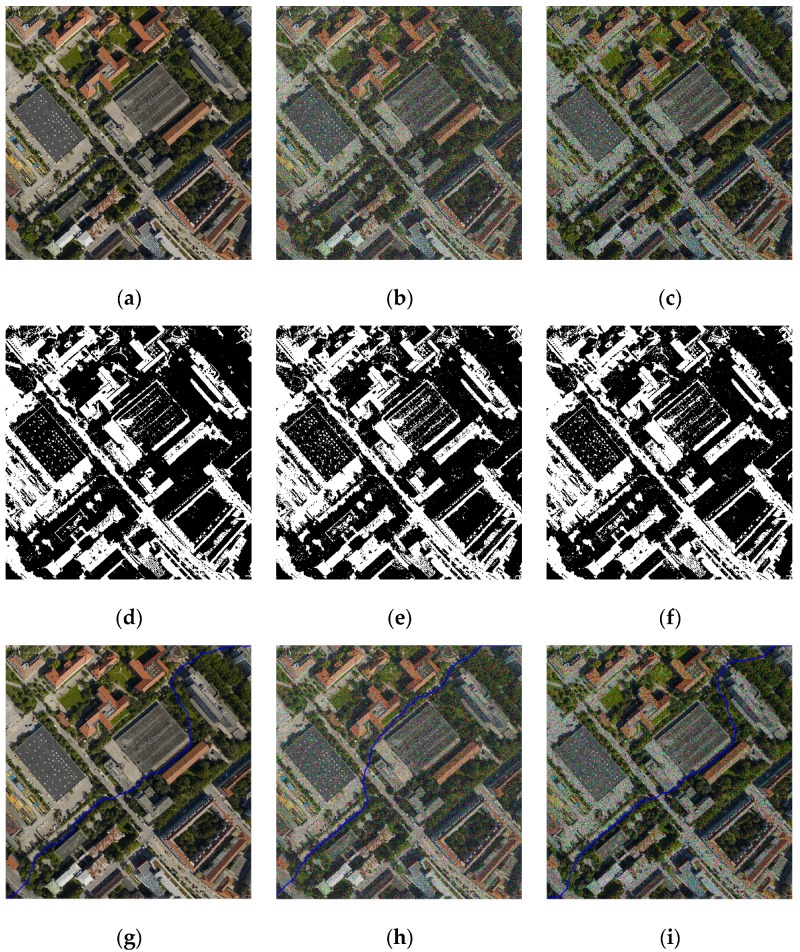
Experimental results of suppressing noise: (**a**) image without noise; (**b**) image with Gaussian noise; (**c**) image with Gamma noise; (**d**) segmentation result of (**a**); (**e**) segmentation result of (**b**); (**f**) segmentation result of (**c**); (**g**)seamline based on (**d**); (**h**) seamline based on (**e**), (**i**) seamline based on (**f**).

In order to test the performance of suppressing noise, we detected seamlines in the images perturbed with Gaussian noise and Gamma noise. Figure 9a shows the original image. Figure 9b,c are the images perturbed with Gaussian noise and Gamma noise. Figure 9d–f are the results of segmentation using the PKGC algorithm. The PKGC algorithm is very strong to suppress noise. As Figure 9e,f shows, obvious objects are segmented effectively. Thus, the seamlines based on the BRs bypass the obvious objects, as Figure 9h,i show. Correspondingly, the method we proposed in this paper is robust and stable to suppressing noise.

### 3.5. Experiment of Remote Sensing Image Mosaicking

**Figure 10:**
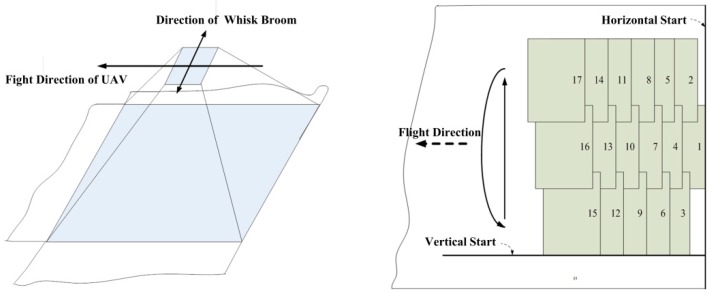
Obtaining remote sensing image set.

The mosaicking dataset is captured by an aerial digital camera integrated in the airborne photoelectric platform. We use the unmanned aerial vehicle (UAV)-equipped airborne photoelectric platform to take images by whisk broom for a ground area. Figure 10 shows the process of obtaining the remote sensing image set. The experimental weather conditions are clear, the flight altitude AGL is 2.1 km, the flight velocity is 30 m/s. The image is captured by an aerial visible light camera, the resolution is 3288 × 2192 pixels, the pixel size is 0.5 μm, the focal length f = 130.2 mm.

**Figure 11:**
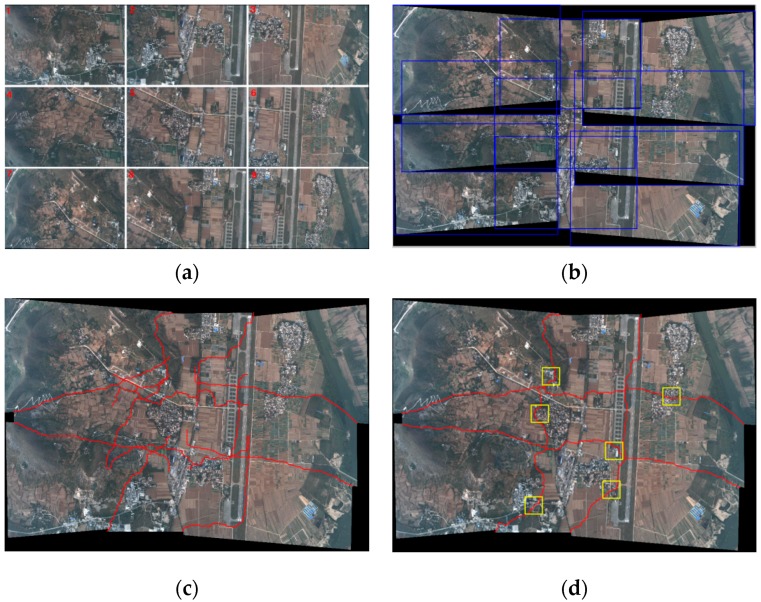

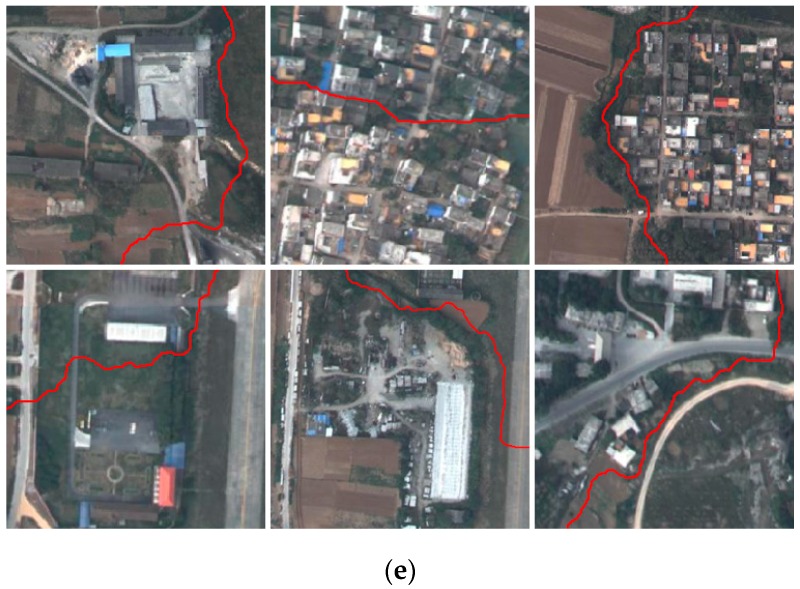
Experimental results of image mosaicking: (**a**) the remote sensing image set; (**b**) positional relations and overlapping regions; (**c**) seamlines in two-image overlap; (**d**) optimal seamlines; (**e**) the details of yellow rectangles in (**d**).

Figure 11a exhibits the remote sensing image dataset. Figure 11b gives the positional relations and overlapping regions between image sequences and the blue rectangle is the edge of each image. Figure 11c shows the seamlines detected in each overlapping region of the two images. Figure 11d are the optimal seamlines using the method we proposed in Section 2.4. Figure 11e shows the enlarged details of the yellow rectangle regions in Figure 11d. The detailed picture shows that the seamlines can effectively avoid the significant target, and pass through the background regions with a smooth texture, such as road, grass, and so on. The seamlines can provide a strong guarantee for the follow-up mosaicking. The comparison of seamlines detected in each two-image overlapping (TIO) region and multi-image overlapping (MIO) region is shown in Table 2. We can see that the optimal method for multi-image seamline detection proposed in Section 2.4 can determine a seamline with fewer pixels and takes less time, only 69.16% of TIO. Meanwhile, the optimal method can effectively resolve the problem that the seamline passes through the obvious objects near the edge of the overlapping region.

**Figure 12:**
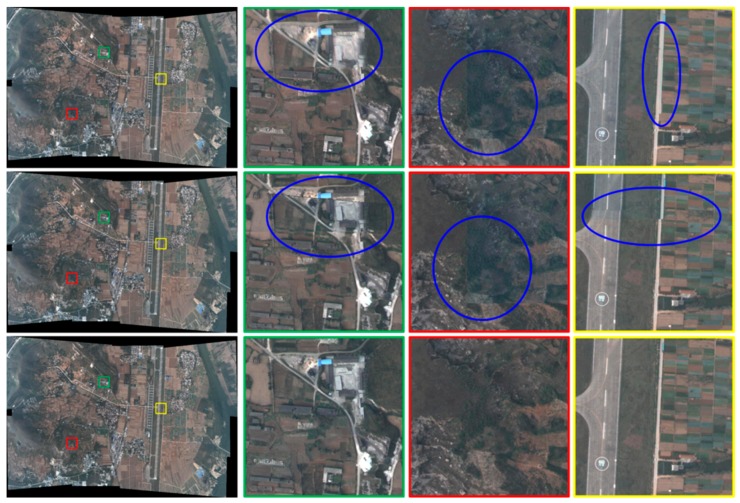
Comparison of different mosaicking methods: the first row is the pixel fusion method; the second row is the two-image overlap seamlines method and the third is the multi-image overlap seamlines method.

We mosaic this dataset by the pixel fusion method, two-image overlap seamlines, and the multi-image overlap seamlines method. Figure 12 shows the compare of mosaicking results by different methods, the first row of which is the result and details by pixel fusion method. The second row is the result based on two-image overlap seamlines and the third row shows the result and details based on the multi-image overlap seamlines. When using the pixel fusion method, the ghost effect will exist in the regions that the corresponding pixels are misaligned. The uneven brightness also exists when the brightness differences are too large. When using the two-image seamlines method, there will be the geometrical dislocations in the misaligned regions and uneven brightness in the regions of the large brightness difference, showing the obvious “seam”. The multi-image overlap seamlines method can solve the above problems effectively. There are no obvious seams, no geometric misalignments, no ghosting, and a high quality of reproduction in the panoramic mosaicking image.

The proposed method is effective and excellent to determine seamlines for remote sensing image mosaicking. However, there is still room for improvement in this method. When the size of the objects is too small and the color is very close to the background, such as black cars in the shadow of buildings, they cannot be extracted. Moreover, although the time-consumption is greatly reduced compared with other methods, it is not sufficient for real-time processing. These issues will be solved in future research.

## 4. Conclusions

We proposed a novel method to determine the seamline based on PKGC segmentation and the combined cost for remote sensing image mosaicking. In this method, we segment the input images by the PKGC algorithm and determine the BRs and ORs based on the result of segmentation firstly. Then the global cost containing the color difference, MSMG constraint, and texture difference is weighted by BRs. Finally, the seamline is determined in the weighted cost from the start point to the end point. Dijkstra’s shortest path algorithm is used to optimize the seamline at the pixel level. The BRs can automatically be obtained by PKGC segmentation in this method. Furthermore, the combined cost can indicate the image information accurately. The new method for multi-image seamline detection can effectively resolve the problems in multi-image mosaicking. Experimental results demonstrate the effectiveness and superiority of the proposed method. Seamlines go within roads or grass and successfully bypass the obvious objects. The performance of the proposed method is much better and faster than Pan’s method. Moreover, this method is particularly suitable for images with objects of larger size. The proposed method is effective and shows potential for remote sensing image mosaicking. The mosaicking image based on the seamlines using the proposed method can satisfy the requirements of both field of view and resolution.

## Figures and Tables

**Table 1:** Comparison of different methods.

Image Set	Items Method	Number of Pixels in Seamline	Number of Obvious Objects Passed Through	Processing Time (s)
1	Chon’s	3694	2 buildings	835.63
	Li’s	3546	2 buildings and 1 car	58.28
	Pan’s	3975	5 buildings	37.20
	The proposed	4158	None	10.87
2	Chon’s	1460	1 building	145.21
	Li’s	1563	4 buildings	25.42
	Pan’s	2254	6 buildings	13.76
	The proposed	1911	None	8.42

**Table 2:** Comparison of seamlines.

Number of Image Overlapping Region	Type of Seamline	Number of Pixels in Seamline	Number of Obvious Objects Passed Through	Processing Time (s)
1-2	TIO	2916	1	2.19
	MIO	2277	0	1.68
1-4	TIO	3302	0	2.10
	MIO	2897	0	1.58
2-3	TIO	3255	0	1.92
	MIO	1863	0	1.42
2-5	TIO	3012	2	1.92
	MIO	1986	0	1.49
3-6	TIO	3706	1	2.52
	MIO	3163	1	2.01
4-5	TIO	3300	0	1.95
	MIO	1646	0	1.41
4-7	TIO	3693	2	1.58
	MIO	2124	1	1.06
5-6	TIO	2755	1	1.96
	MIO	1598	0	1.50
5-8	TIO	3621	1	1.48
	MIO	2320	0	0.95
6-9	TIO	4270	1	2.62
	MIO	2747	0	2.08
7-8	TIO	4114	0	2.56
	MIO	2793	0	2.03
8-9	TIO	3711	0	2.23
	MIO	3396	0	1.77
